# Nano-ZnO-Crosslinked Pectin/CMC Film with Enhanced Hydrophobicity and UV-Blocking for Blueberry Packaging

**DOI:** 10.3390/polym18111316

**Published:** 2026-05-27

**Authors:** Xu Dong, Haijuan Zhu, Jianhua Zheng, Zhongliang Wang, Sihang Zhang

**Affiliations:** 1 College of Light Industry and Textile, Qiqihar University, 42 Culture Street, Qiqihar 161006, China; jianhua0904@163.com (J.Z.); paperwzl@163.com (Z.W.); 2 Engineering Research Center for Hemp and Product in Cold Region of Ministry of Education, Qiqihar 161006, China; 3 Green Food Development Center of Qiqihar City, Qiqihar 161000, China; 15636259288@163.com; 4 School of Food Science and Engineering, Hainan University, Haikou 570228, China

**Keywords:** pectin/CMC, ZnO, hydrophobicity, UV blocking, packaging

## Abstract

Developing a biodegradable film with integrated mechanical robustness and multifunctionality remains a significant challenge for sustainable food packaging. Herein, a pectin/carboxymethyl cellulose composite film (PNZ_x_C) incorporated with zinc oxide nanoparticles (ZnO) was fabricated via a solution casting method to achieve the synergistic enhancement of structural and functional properties. ZnO exhibits dual functionality within the polymer matrix, serving both as a reinforcing filler and as a coordination interaction node via interactions with carboxyl groups. At an optimal loading, the PNZ_2_C film demonstrates a uniform dispersion of nanoparticles, facilitating the development of a dense network structure and enhancing intermolecular interactions. Consequently, the film showed reduced water vapor and oxygen permeability, attributable to the formation of tortuous diffusion pathways, together with increased surface hydrophobicity and a significantly improved tensile strength of 25.4 MPa. Enhanced thermal stability and excellent UV-blocking performance were also achieved. Notably, the optimized film demonstrated superior preservation performance in blueberry storage, effectively reducing moisture loss and delaying quality deterioration compared with the control. These findings provide new insights into the structure–property relationships of ZnO–polysaccharide nanocomposite systems and highlight a viable strategy for designing high-performance, biodegradable packaging materials with integrated multifunctionality.

## 1. Introduction

Fresh fruits are highly vulnerable to physiological degradation and microbial spoilage during postharvest storage, resulting in substantial quality loss and shortened shelf life. Such deterioration not only negatively affects the sensory and nutritional attributes of fruits but also increases the potential food safety risks to consumers [1,2]. Food packaging materials therefore play an indispensable role in maintaining product quality throughout storage, transportation, and commercial distribution [3,4]. Among the various packaging systems, functional packaging films are particularly important because they can establish a protective microenvironment that effectively limits the influence of external factors, including oxygen, water vapor, ultraviolet irradiation, and microbial contamination [5]. Consequently, these films contribute significantly to preserving freshness and extending the storage stability of fresh produce. Despite their excellent mechanical performance and low production cost, conventional petroleum-derived plastic packaging materials have raised growing environmental concerns due to their resistance to degradation, long-term accumulation in ecosystems, and dependence on finite fossil resources [6,7]. In recent years, global strategies aimed at carbon neutrality and circular bioeconomy development have further accelerated research interest in biodegradable and renewable packaging materials. Therefore, the development of environmentally friendly alternatives capable of replacing traditional petroleum-based plastics has emerged as a critical research focus in the field of sustainable food packaging.

Biodegradable films derived from natural, biocompatible, and renewable biopolymers have gained increasing attention as viable candidates, particularly for food-contact applications [8,9,10]. Among these, polysaccharides including cellulose, starch, lignin, and pectin have been extensively investigated due to their abundance, biocompatibility, and film-forming capability [11,12,13,14]. Pectin, a plant-derived polysaccharide rich in carboxyl groups, exhibits excellent gelling ability and favorable oxygen barrier properties, making it an attractive material for packaging film [15,16]. Nevertheless, its practical application is hindered by intrinsic limitations, including brittleness, rigidity, high water sensitivity, and poor moisture resistance. To overcome these drawbacks, blending pectin with other biopolymers has been widely adopted. Carboxymethylcellulose (CMC), a water-soluble cellulose derivative, offers superior flexibility, mechanical strength, and biocompatibility [17,18,19]. Due to their anionic nature, pectin and CMC can form physically crosslinked networks through intermolecular interactions, resulting in improved structural stability and mechanical performance. Despite these advantages, such biopolymer-based films still suffer from insufficient barrier properties and limited multifunctionality compared with conventional plastics, restricting their large-scale application.

The incorporation of inorganic nanomaterials into polymer matrices has been widely recognized as an effective strategy for enhancing the physicochemical and functional properties of polymer-based materials [20,21,22,23]. The incorporation of inorganic nanomaterials into polymer matrices has been widely recognized as an effective strategy for enhancing the physicochemical and functional properties of polymer-based materials. Representative studies on nanoparticle-incorporated polysaccharide films for food preservation are summarized in Table 1. Among the various inorganic nanofillers investigated, ZnO nanoparticles (nano-ZnO) have attracted particular attention because of their excellent stability, UV-shielding capability, and antimicrobial activity [24,25,26]. Beyond serving as a reinforcing nanofiller, ZnO can interact with the pectin/CMC matrix through coordination interactions with carboxyl groups and hydrogen bonding. Such interactions can further regulate the polymer network structure and improve interfacial compatibility within the composite system [27,28]. At appropriate nanoparticle loadings, these interactions contribute to enhanced interfacial compatibility and improved overall film performance. However, excessive incorporation of ZnO may lead to nanoparticle aggregation and interfacial incompatibility within the polymer matrix. As a consequence, microvoids, structural discontinuities, and localized stress concentration sites may develop within the composite films [29]. More importantly, the ability of ZnO to regulate structural defects within the pectin/CMC composite system may critically influence the structural integrity and long-term stability of the resulting films. Nevertheless, how these ZnO-mediated structural regulation effects influence fresh-food preservation performance remains poorly understood. To address this issue, blueberries were selected as a model fruit system because of their high nutritional value and pronounced perishability [30,31]. Due to their thin epidermal structure and high respiration rate, blueberries are extremely vulnerable to moisture loss, tissue softening, oxidative deterioration, and microbial contamination during ambient storage, which ultimately results in rapid quality degradation and reduced shelf life. Therefore, blueberries provide an ideal model for evaluating the practical preservation effectiveness of active packaging materials.

Based on this model system, the influence of in situ coordination interactions induced by ZnO nanoparticles on the structural and functional properties of pectin/CMC composite films was systematically investigated. The film was fabricated via a solution-casting method, and their physicochemical properties, including hydrophobicity, water vapor and oxygen barrier properties, mechanical performance, and UV-shielding capability, were systematically evaluated. Furthermore, the microstructure, intermolecular interactions, and thermal stability of the film were characterized using scanning electron microscopy, Fourier transform infrared spectroscopy, X-ray diffraction, and thermogravimetric analysis. In addition, the application potential of the developed nanocomposite film was assessed in the preservation of blueberries. This work provides new insights into the structure–property relationships of inorganic–organic hybrid films and offers a feasible strategy for designing high-performance, biodegradable packaging materials with integrated functionality.

## 2. Experimental

### 2.1. Materials and Chemicals

Sodium carboxymethyl cellulose (CMC, viscosity: 1000–1400 mPa·s), zinc oxide nanoparticles (nano-ZnO, particle size ≤ 100 nm, purity ≥ 99%), and pectin (galacturonic acid content ≥ 74.0%, dry basis) were supplied by Aladdin Industrial Corporation (Shanghai, China). Glycerol (C_3_H_8_O_3_, analytical reagent grade, ≥99.0%) and ethanol (C_2_H_5_OH, analytical reagent grade, ≥99.7%) were purchased from Sinopharm Chemical Reagent Co., Ltd. (Shanghai, China). Deionized water was prepared in the laboratory, whereas all chemicals were directly used as received without any further purification.

### 2.2. Preparation of Nano-ZnO Solution

A predetermined amount of dried nano-ZnO was first dispersed in 98 mL of deionized water, after which glycerol (0.6 g) was introduced as a plasticizer. The mixture underwent sonication (25 kHz, 250 W) for 30 min at approximately 35 °C to promote effective dispersion, after which it was stirred at 800 rpm for 30 min until a homogeneous nano-ZnO dispersion was obtained for subsequent use.

### 2.3. Preparation of Film

**Figure 1:**
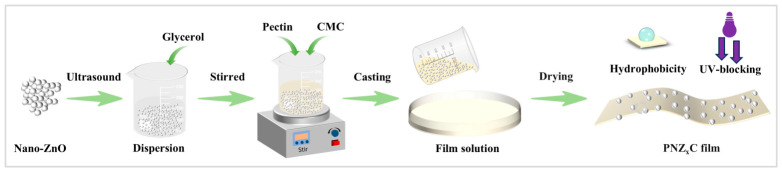
Schematic illustration of the fabrication process of the PNZ_x_C film.

Pectin (1 g) was introduced into the prepared ZnO dispersion and stirred for 1 h at room temperature until fully dissolved, followed by the addition of CMC (1 g) with stirring at 600 rpm for 30 min. The mixture was subsequently heated to 60 °C and maintained under continuous stirring until a homogeneous film-forming solution was formed. The solution volume was adjusted to 50 mL and subsequently cast into Petri dishes (15 cm in diameter), followed by drying in a hot-air oven at 30 °C for 48 h to ensure uniform film thickness. The dried film was carefully removed and equilibrated at 25 °C and 50% relative humidity for at least 48 h before characterization. The film was labeled as PNZ_x_C, where x represents the mass fraction (%) of nano-ZnO relative to the total mass of pectin and CMC. The preparation process of the PNZ_x_C film is schematically presented in Figure 1.

### 2.4. Characterization

The surface and cross-sectional morphologies of the prepared films were comprehensively characterized using scanning electron microscopy (SEM; JSM-7800F, JEOL, Tokyo, Japan). For surface morphology analysis, the film samples were directly mounted onto the specimen holder and sputter-coated with a thin layer of gold to enhance electrical conductivity prior to observation. For cross-sectional characterization, the dried films were cryogenically fractured in liquid nitrogen to obtain clean fracture surfaces and subsequently coated with gold before imaging. SEM observations were carried out at an accelerating voltage of 2 kV to investigate the surface topography, internal microstructure, and interfacial characteristics of the films. Fourier transform infrared (FTIR) spectra were acquired using a PerkinElmer spectrometer (USA) within the wavenumber range of 4000–400 cm^−1^ at a spectral resolution of 1 cm^−1^ with 32 scanning cycles using the KBr pellet technique. Crystalline structures of the samples were analyzed by X-ray diffraction (XRD) using an XRD-6100 diffractometer (Shimadzu, Kyoto, Japan) equipped with Cu Kα radiation operated at 40 kV and 30 mA. Diffraction patterns were recorded over a 2θ range of 10–60° at a scanning speed of 10° min^−1^. Thermal stability was further investigated by thermogravimetric analysis (TGA) under a nitrogen atmosphere from 25 to 600 °C at a heating rate of 20 °C min^−1^ to evaluate the thermal degradation behavior of the films.

### 2.5. Mechanical Properties

The tensile properties of the film were determined using a universal testing machine (XLW-PC, Labthink, Jinan, China). The film samples were prepared as rectangular strips (50 mm × 20 mm) and equilibrated at 25 °C and 50% relative humidity for 48 h. The crosshead speed was set at 25 mm/min. At least three replicates were tested for each sample, and the average values were reported.

### 2.6. Gas Barrier Properties

The water vapor transfer rate (WVTR) was measured by using a water vapor permeability tester (W3/010, Labthink, Jinan, China), and the film test was performed at 25 ± 2 °C and 90% RH. The oxygen transmission rate (OTR) was measured using a gas permeability analyzer (BASIC201, Labthink, Jinan, China) under standard testing conditions at 25 ± 2 °C and 50% relative humidity (RH), with an oxygen partial pressure difference of 0.1 MPa across the film. At least three replicates were tested for each sample, and the mean values were reported. Statistical analyses were performed using Origin software (Version 2023, OriginLab Corporation, Northampton, MA, USA). Differences between groups were analyzed using an independent-samples t-test or one-way analysis of variance. A *p*-value of less than 0.05 was considered statistically significant.

### 2.7. UV-Blocking Performance

The UV–visible transmittance spectra of the film were collected using a UV–Vis spectrophotometer (UH5300, Hitachi, Tokyo, Japan) over a wavelength range of 200–800 nm. The UV-blocking efficiency in the UVB (280–320 nm, integrated wavelength range of 40 nm) and UVA (320–400 nm, integrated wavelength range of 80 nm) regions was calculated as follows:

(1) $$\mathrm{UVB}\;\mathrm{blocking}\;(\%)=(1-\frac{\int_{280}^{320}T\left(\lambda \right)d\left(\lambda \right)}{\int_{280}^{320}d\left(\lambda \right)})\;\times \;100$$

(2) $$\mathrm{UVA}\;\mathrm{blocking}\;(\%)=(1-\frac{\int_{320}^{400}T\left(\lambda \right)d\left(\lambda \right)}{\int_{320}^{400}d\left(\lambda \right)})\;\times \;100$$

### 2.8. Water Contact Angle

Surface wettability was evaluated using a contact angle analyzer (DSA100, Krüss, Hamburg, Germany). A 2 μL droplet of ultrapure water was carefully dispensed onto the film surface, and the contact angle was measured after 10 s. All tests were performed at room temperature.

### 2.9. Blueberry Preservation Performance

Fresh blueberries obtained from a local market were randomly assigned to different groups. In each group, four fruits were coated with different films (PNZ_0.2_C, and PE film), while an untreated group served as the control. All blueberry samples were stored at 24–26 °C and 25% relative humidity. The preservation performance was assessed by monitoring the mass loss over time, which was calculated according to the following equation:

(3) $$\mathrm{Mass}\;\mathrm{loss}\;(\%)=\frac{M_{s}-M_{t}}{M_{s}}\times 100$$

where M_s_ and M_t_ represent the initial and final mass, respectively.

## 3. Results and Discussion

### 3.1. Structure Characterization of PNZ_x_C Film

**Figure 2:**
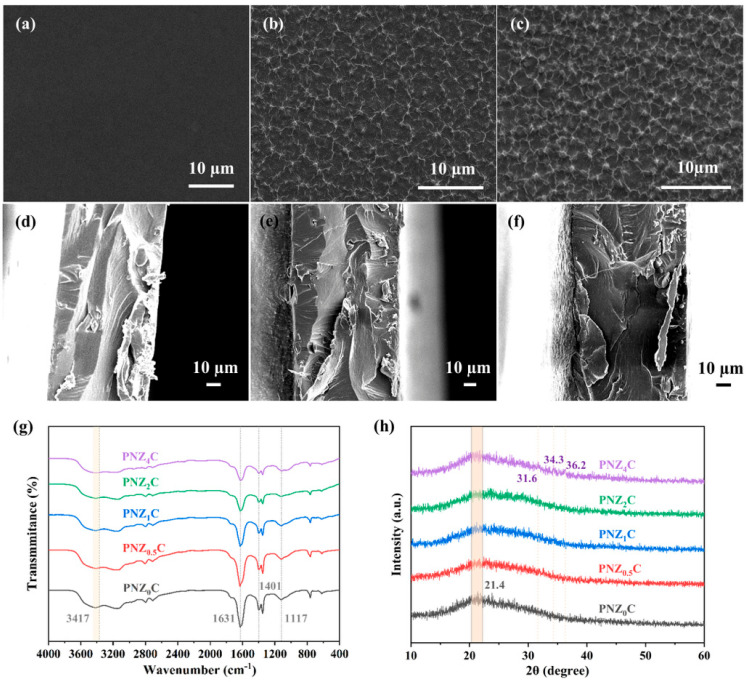
Surface and cross-sectional SEM images of PNZ_0_C (**a**,**d**), PNZ_2_C (**b**,**e**), and PNZ_4_C (**c**,**f**); FTIR spectrum (**g**), and XRD patterns (**h**) of the PNZ_x_C film.

The surface and cross-sectional morphologies of PNZ_x_C films with varying nano-ZnO contents were characterized by SEM. As shown in Figure 2a–c, the ZnO-free control film (PNZ_0_C) exhibited a smooth, dense structure with no observable pores and a uniform, continuous surface, indicative of good compatibility between pectin and CMC and the formation of a homogeneous film matrix. The incorporation of nano-ZnO led to a pronounced change in the surface morphology of the composite film. At low Zn content (PNZ_2_C), the nanoparticles were uniformly dispersed within the polymer matrix, and no visible cracks, phase separation, or major structural discontinuities were observed, indicating that the polymer network remained intact and homogeneous. This suggests that ZnO was effectively embedded within the polymer network and interacted with the matrix, contributing to enhanced structural compactness. However, at higher ZnO content (PNZ_4_C), the film surface became heterogeneous, with evident nanoparticle agglomeration and localized rough or fractured regions. This behavior can be attributed to strong interparticle interactions, which reduce nanoparticle dispersion and disrupt film uniformity. Cross-sectional observations further corroborate these findings, as shown in Figure 2d–f. An appropriate ZnO content results in a more compact internal structure. The film (PNZ_2_C) exhibited a continuous morphology without apparent phase separation. This behavior may be attributed to coordination interactions between Zn^2+^ ions and carboxyl groups in the polysaccharide chains, which enhance intermolecular interactions and facilitate the establishment of a tightly packed network structure. In contrast, excessive ZnO (PNZ_4_C) led to particle aggregation and localized microcracks, indicating reduced dispersion and the introduction of structural defects. These results indicate that ZnO exerts both reinforcing and detrimental effects on the film system, where its dispersion state plays a critical role in determining the final microstructure [37].

FTIR was used to further elucidate the molecular interactions within the composite film. All samples exhibited characteristic polysaccharide absorption bands in the range of 4000–400 cm^−1^ (Figure 2g). The broad band at 3417 cm^−1^ was attributed to O–H stretching modes, indicating extensive hydrogen bonding within the system. With increasing ZnO content, this band showed slight shifts and intensity variations, suggesting that ZnO incorporation modifies the hydrogen-bonding network. The bands at 1631 cm^−1^ and 1401 cm^−1^ were assigned to asymmetric and symmetric stretching modes of carboxylate (–COO^−^) groups, respectively [38]. The shifts in band position and variations in intensity suggest coordination between nano-ZnO and carboxyl functionalities in pectin and CMC. In addition, the bands at approximately 1117 cm^−1^, assigned to C–O–C and C–O stretching vibrations, remained largely unchanged, indicating that the polysaccharide backbone structure is preserved. FTIR analysis confirmed that ZnO nanoparticles were successfully incorporated into the pectin/CMC system and strengthened intermolecular interactions via hydrogen bonding and coordination with carboxyl groups, thereby facilitating the development of a core network architecture.

XRD analysis further clarified the crystalline structure of the composite films. As shown in Figure 2h, the pectin/CMC matrix film (PNZ_0_C) displayed a broad diffraction peak centered at a 2θ value of approximately 21.4°, indicative of a predominantly amorphous structure characteristic of polysaccharide-based materials [39,40]. Following the incorporation of nano-ZnO, the diffraction pattern exhibited marked changes. For the PNZ_4_C film, distinct reflections at approximately 2θ = 31.6°, 34.3°, and 36.2°, characteristic of the hexagonal wurtzite phase of ZnO, indicate the successful inclusion of ZnO within the polymer matrix. Moreover, the intensity of these peaks increased with increasing ZnO content, indicating enhanced crystallinity [41]. This effect is likely due to the nucleating role of nano-ZnO, which promotes the formation of locally ordered regions within the polymer matrix. Meanwhile, subtle variations in the broad peak around 21.4° suggest that ZnO influences the arrangement of pectin/CMC molecular chains without disrupting the overall amorphous structure. Thus, although the films remain predominantly amorphous, the degree of local ordering is enhanced. The XRD analysis suggests that ZnO-modified films exhibit enhanced crystallinity and molecular ordering, thereby improving their mechanical strength and barrier performance.

### 3.2. Mechanical Performance, Surface Hydrophobicity, Barrier Properties, and Thermal Stability

**Figure 3:**
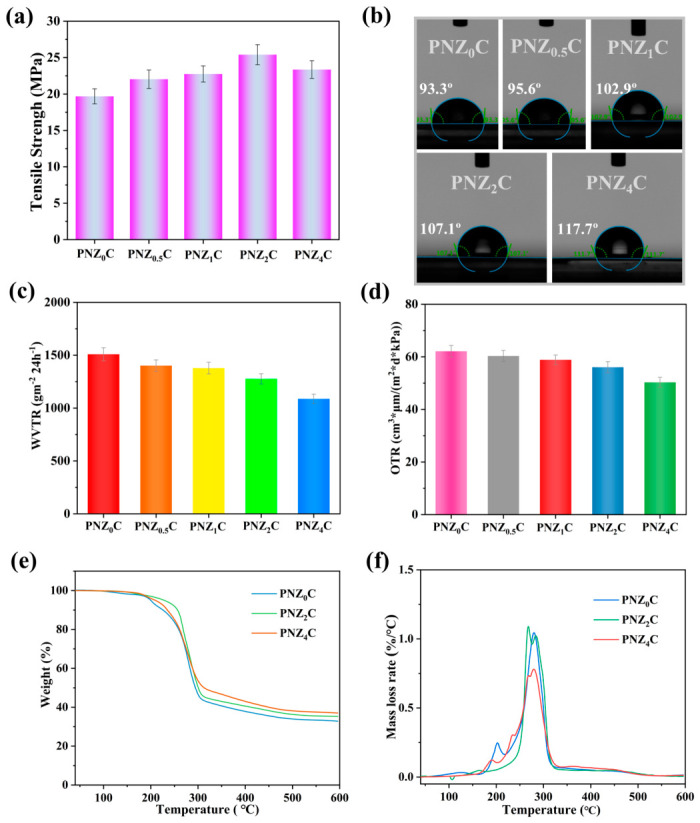
Tensile strength (**a**), surface hydrophobicity (**b**), WVTR (**c**), and OTR (**d**) of the PNZ_x_C film; weight loss curves (**e**) and mass loss rate curves (**f**) of the PNZ_0_C, PNZ_2_C, and PNZ_4_C films.

The mechanical properties of the film were assessed via tensile strength measurements (Figure 3a). The tensile strength initially increased and subsequently decreased with increasing ZnO content. The control film (PNZ_0_C) exhibited a tensile strength of 19.6 MPa, whereas the incorporation of nano-ZnO significantly enhanced the tensile strength to 25.4 MPa for PNZ_2_C, indicating improved mechanical performance. This enhancement can be primarily attributed to the incorporation of ZnO within the polymer matrix. Specifically, the ionic cross-linking interactions between Zn^2+^ ions and carboxyl groups serve as physical crosslinked nodes, thereby facilitating stress transfer and reinforcing the overall network structure. In addition, secondary interactions, including hydrogen bonding and coordination between Zn^2+^ ions and the pectin/CMC chains, promoted the formation of a more stable crosslinked network, thereby enhancing the structural integrity of the film. However, further increasing the ZnO content (PNZ_4_C) resulted in a decrease in tensile strength to 23.6 MPa, which was likely attributed to nanoparticle agglomeration, leading to stress concentration and reduced structural homogeneity. These findings suggest that the mechanical performance of the films is primarily governed by the homogeneous dispersion of nano-ZnO and the formation of an optimized crosslinked network [42].

The surface hydrophobicity of the films was assessed by contact angle measurements (Figure 3b). The contact angle increases progressively with increasing ZnO content, indicating enhanced hydrophobicity. The control film (PNZ_0_C) exhibited a contact angle of approximately 93.3°, whereas PNZ_4_C reached approximately 111.7°, representing a substantial increase in hydrophobicity. This behavior is governed by the interplay between ZnO incorporation-induced surface roughness and the shielding of hydrophilic –OH and –COO^−^ groups via intermolecular interactions. Moreover, the intrinsic inorganic nature of ZnO contributes to lowering the surface energy, further enhancing the hydrophobic performance [43,44].

The barrier properties of the film were evaluated in terms of WVTR (Figure 3c) and OTR (Figure 3d). Both WVTR and OTR decreased with increasing ZnO content, indicating improved resistance to water vapor and oxygen permeation. The PNZ_2_C sample exhibited significantly lower water vapor transmission rate (WVTR) and oxygen transmission rate (OTR) values of 1276.6 g/(m^2^·24 h) and 50.3 m^3^·μm/(m^2^·d·kPa), respectively (*p* < 0.05). This improvement can be attributed to the incorporation of ZnO, which creates a more tortuous diffusion pathway within the polymer matrix, thereby increasing the diffusion distance and reducing the transmission rate of oxygen and water vapor molecules. In addition, the interactions between ZnO and the polymer chains may decrease the free volume and restrict polymer chain mobility, resulting in a denser microstructure. At an appropriate nanoparticle loading, the uniform dispersion of ZnO further enhances interfacial interactions and structural compactness, thereby improving the oxygen and water vapor barrier properties of the composite films [45]. Notably, no significant deterioration in barrier properties was observed at higher ZnO content (PNZ_4_C), suggesting that ZnO remained relatively well-dispersed within the studied concentration range.

The composite films underwent TGA to assess their thermal stability (Figure 3e,f), exhibiting a typical three-stage thermal degradation profile. The initial weight loss (30–150 °C) resulted from the evaporation of free and bound water, followed by a major degradation stage (200–350 °C), corresponding to the decomposition of pectin and CMC molecular chains. The final stage above 350 °C was associated with carbonization and residual char formation. Compared with PNZ_0_C, the ZnO-containing film showed higher onset decomposition temperatures and increased residual mass at elevated temperatures. At approximately 595–600 °C, the residual mass increased from 32.85% (PNZ_0_C) to 35.26% (PNZ_2_C) and 37.02% (PNZ_4_C). Furthermore, the DTG curves revealed a gradual decrease in the maximum weight loss rate with increasing ZnO content, indicating suppression of the thermal degradation process. This enhancement can be attributed to the synergistic effects of ZnO acting as a thermal barrier and the potential coordination interactions between ZnO and the polysaccharide chains. These interactions strengthen the network structure and increase the energy required for thermal degradation, thereby improving the thermal stability and residual mass at elevated temperatures [46].

Overall, nano-ZnO incorporation leads to simultaneous improvements in mechanical strength, hydrophobicity, barrier performance, and thermal stability of the composite films. These enhancements arise from an integrated mechanism involving inorganic reinforcement, intermolecular crosslinked, and tortuous diffusion pathways, highlighting the critical role of ZnO in modulating polymer matrix dispersion and interactions.

### 3.3. UV-Blocking Properties

**Figure 4:**
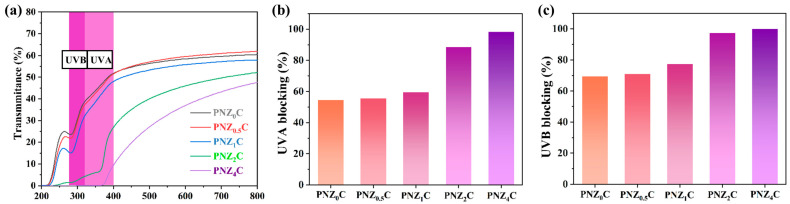
Light transmittance spectra (**a**), UVA blocking rate (**b**), and UVB blocking rate (**c**) of the PNZ_x_C film.

The UV–Vis transmission spectrum of the composite films is displayed in Figure 4a. The control film (PNZ_0_C) exhibited partial transmittance in the UV region, indicating limited UV-shielding capability. As ZnO nanoparticle content increased, the transmittance of the films decreased markedly in both the UVB (280–320 nm) and UVA (320–400 nm) regions, demonstrating improved UV-shielding performance. Notably, the PNZ_2_C and PNZ_4_C films showed almost zero UV transmittance across the 280–320 nm range, demonstrating outstanding UV-shielding capability.

Quantitative analysis further confirmed this trend. The UVB shielding efficiency increased from 69.4% for PNZ_0_C to nearly 100% for the ZnO-containing films (Figure 4c), while the UVA shielding efficiency increased from 54.4% to 98.2% (Figure 4b). In particular, PNZ_2_C exhibited a UVB shielding efficiency of 97.4% and a UVA shielding efficiency of 88.6%, highlighting the strong UV absorption capability of nano-ZnO, particularly in the UVB region. This enhancement can be attributed to the strong UV absorption and light-scattering properties of ZnO nanoparticles, which effectively reduced UV transmittance through the composite films [42]. When well-dispersed within the polymer matrix, nano-ZnO not only improved UV absorption but also reduced UV transmittance through light scattering effects. However, the enhanced UV-shielding performance was accompanied by reduced transparency, suggesting a trade-off between UV protection and optical clarity. This effect is mainly attributed to increased light scattering induced by the dispersion of ZnO within the polymer matrix. Nevertheless, the composite films retained acceptable transparency and remained suitable for packaging applications. In particular, the film containing a moderate ZnO loading (PNZ_2_C) exhibited significantly improved UV-blocking performance, demonstrating its strong potential for food-packaging applications.

### 3.4. Application in the Preservation of Blueberries

**Figure 5:**
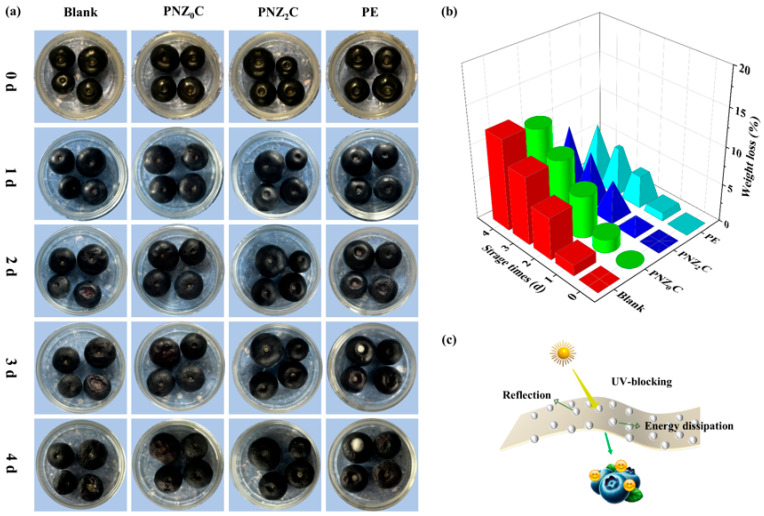
Preservation and monitoring images (**a**), mass loss of blueberries packaged with blank, PNZ_0_C, PNZ_2_C, and PE films during 4 days of storage (**b**), and schematic illustration of the UV-shielding capability of the PNZ_2_C film (**c**).

Fresh blueberries were used as a model system to evaluate the preservation performance of the composite film. Blueberries are highly perishable fruits that rapidly deteriorate under ambient storage conditions due to moisture loss, tissue softening, and microbial spoilage. To assess the short-term preservation performance of the different films while ensuring that the blueberries remained in an acceptable consumable state during storage, the samples were evaluated over 4 days. As shown in Figure 5a, the evolution of blueberry appearance throughout storage was evaluated across the untreated (blank), PNZ_0_C, PNZ_2_C, and commercial polyethylene (PE) film groups under identical conditions. After 4 days of storage, all samples exhibited varying degrees of shrinkage due to moisture loss; however, distinct differences in decay behavior were observed among the groups. Blueberries packaged with PE films showed noticeable mold growth and deterioration, whereas those packaged with composite films, particularly PNZ_2_C, maintained better overall appearance and freshness.

The mass loss of blueberries during storage is presented in Figure 5b. In all groups, mass loss increased progressively over time; however, the extent of mass loss varied significantly depending on the packaging treatment. The untreated group exhibited the highest mass loss (12.1%), indicating severe moisture loss. In contrast, samples packaged with composite films showed reduced mass loss, demonstrating their effectiveness in limiting water evaporation. Among them, PNZ_2_C exhibited a lower mass loss rate of 8.8%, indicating superior preservation performance compared to PNZ_0_C. Although the PE-packaged samples showed the lowest mass loss (7.2%), they suffered from severe spoilage, suggesting that minimizing moisture loss alone is insufficient to ensure effective preservation.

The enhanced preservation performance of ZnO-containing composite films can be attributed to multiple synergistic effects [47]. The reduced water vapor permeability effectively inhibits moisture loss, thereby limiting moisture loss during storage. The decreased oxygen permeability slows respiration and metabolic activity, consequently reducing substrate consumption. In addition, the improved UV-shielding capability of the films suppresses photooxidation reactions (Figure 5c). The PNZ_2_C film exhibited the most balanced preservation performance, effectively delaying the deterioration of blueberry quality through the combined effects of improved barrier properties and functional performance. These results highlight its strong potential for application in food packaging.

## 4. Conclusions

In this study, the PNZ_x_C film was formed by solution casting, and its structure–property relationships were systematically explored. The results demonstrate that ZnO plays a dual role in the composite system, acting not only as a reinforcing filler but also as a coordination crosslinker via interactions with carboxyl groups, thereby modulating the polymer network structure. At an optimal loading level, nano-ZnO is well-dispersed throughout the matrix, leading to the formation of a dense, continuous network. This structural optimization results in significant improvements in mechanical strength, surface hydrophobicity, barrier performance, and thermal stability. Notably, the optimized PNZ_2_C film exhibited excellent UV-shielding capability, with a UVB shielding efficiency of 97.4% and UVA shielding efficiency of 88.6%. Furthermore, the practical applicability of the composite films was validated through blueberry preservation experiments, where the optimized film effectively reduced moisture loss, suppressed spoilage, and maintained fruit quality during storage. Here, we provide insights into the regulation of intermolecular interactions and microstructure in polysaccharide-based nanocomposite films and propose a strategy for developing high-performance, biodegradable, multifunctional packaging materials.

## Figures and Tables

**Table 1:** Recent studies on particle-incorporated biopolymer films for preservation applications.

Film Matrix	Particles	Main Functional Improvement	Preservation Applications	Ref.
Sodium alginate/zein	ZIF-8	Antioxidant (DPPH 85.48%; ABTS 78.24%)	Apricot preservation	[32]
Starch	ZIF-67	Antioxidant capacity (86.93%)	Agaricus bisporus preservation	[31]
Cellulose acetate	Fe/Cu/Al	Oxygen scavenging activity (0.994 day^−1^)	Liquid food packaging	[33]
Chitosan/polyvinyl alcohol	ZnO	UVB-shielding property (99.99%)	Food preservation applications	[34]
Density polyethylene	Nano-ZnO	Antibacterial activity against Pseudomonas fluorescens	Fruit and vegetable packaging	[35]
Cellulose/poly(lactic acid)	Nano-ZnO	UV shielding (92.20% at 320 nm)	Biodegradable packaging applications	[36]

## Data Availability

The data supporting the findings of this study are available from the author upon reasonable request.
